# Review: evolution of GnIH and related peptides structure and function in the chordates

**DOI:** 10.3389/fnins.2014.00255

**Published:** 2014-08-15

**Authors:** Tomohiro Osugi, Takayoshi Ubuka, Kazuyoshi Tsutsui

**Affiliations:** Laboratory of Integrative Brain Sciences, Department of Biology, Center for Medical Life Science, Waseda UniversityTokyo, Japan

**Keywords:** gonadotropin-inhibitory hormone (GnIH), RF-amide peptides, reproduction, evolution, chordates, lamprey, amphioxus

## Abstract

Discovery of gonadotropin-inhibitory hormone (GnIH) in the Japanese quail in 2000 was the first to demonstrate the existence of a hypothalamic neuropeptide inhibiting gonadotropin release. We now know that GnIH regulates reproduction by inhibiting gonadotropin synthesis and release *vi*a action on the gonadotropin-releasing hormone (GnRH) system and the gonadotrope in various vertebrates. GnIH peptides identified in birds and mammals have a common LPXRF-amide (X = L or Q) motif at the C-terminus and inhibit pituitary gonadotropin secretion. However, the function and structure of GnIH peptides are diverse in fish. Goldfish GnIHs possessing a C-terminal LPXRF-amide motif have both stimulatory and inhibitory effects on gonadotropin synthesis or release. The C-terminal sequence of grass puffer and medaka GnIHs are MPQRF-amide. To investigate the evolutionary origin of GnIH and its ancestral structure and function, we searched for GnIH in agnathans, the most ancient lineage of vertebrates. We identified GnIH precursor gene and mature GnIH peptides with C-terminal QPQRF-amide or RPQRF-amide from the brain of sea lamprey. Lamprey GnIH fibers were in close proximity to GnRH-III neurons. Further, one of lamprey GnIHs stimulated the expression of lamprey GnRH-III peptide in the hypothalamus and gonadotropic hormone β mRNA expression in the pituitary. We further identified the ancestral form of GnIH, which had a C-terminal RPQRF-amide, and its receptors in amphioxus, the most basal chordate species. The amphioxus GnIH inhibited cAMP signaling *in vitro*. In sum, the original forms of GnIH may date back to the time of the emergence of early chordates. GnIH peptides may have had various C-terminal structures slightly different from LPXRF-amide in basal chordates, which had stimulatory and/or inhibitory functions on reproduction. The C-terminal LPXRF-amide structure and its inhibitory function on reproduction may be selected in later-evolved vertebrates, such as birds and mammals.

## Introduction

Reproduction is one of the essential mechanisms for life. In vertebrates, the hypothalamic-pituitary-gonadal (HPG) axis is known as the core mechanism regulating reproduction. Gonadotropin-releasing hormone (GnRH) is the key hypothalamic neuropeptide that regulates the HPG axis by stimulating secretion of gonadotropins, i.e., luteinizing hormone (LH) and follicle-stimulating hormone (FSH), from the anterior pituitary. GnRH was first discovered in the brain of mammals at the beginning of 1970s (Matsuo et al., 1971; Burgus et al., 1972), and it was subsequently identified in the brain of non-mammalian vertebrates (King and Millar, 1982; Miyamoto et al., 1982, 1984; Sherwood et al., 1986). On the other hand, until recently no hypothalamic neuropeptide that inhibits gonadotropin release has been identified, although gonadal sex steroids and inhibin can inhibit gonadotropin release.

It became clear that the regulatory mechanism of reproduction is not as simple as it was once considered, since gonadotropin-inhibitory hormone (GnIH), a novel hypothalamic neuropeptide, was found to be involved in the regulation of the HPG axis (Tsutsui et al., 2000). GnIH was originally identified in birds (Tsutsui et al., 2000) and it was subsequently identified in other vertebrates from fish to humans (for reviews, see Ukena and Tsutsui, 2005; Tsutsui and Ukena, 2006; Tsutsui et al., 2006, 2007, 2010a,b, 2012, 2013; Tsutsui, 2009; Tsutsui and Ubuka, 2012). The discovery of GnIH has now changed our understanding about regulation of the reproductive axis fundamentally (for reviews, see Tsutsui et al., 2006, 2007, 2010a,b, 2012, 2013; Tsutsui, 2009; Tsutsui and Ubuka, 2012; Ubuka et al., 2013a).

To investigate the evolutionary origin of GnIH, we identified the orthologous gene of GnIH and mature GnIH peptides in the brain of lamprey, one of the oldest lineage of vertebrates, Agnatha (Osugi et al., 2012). Recently we further identified the ancestral form of GnIH in amphioxus, the most basal chordates (Osugi et al., 2014). These studies suggest that the origin of GnIH-like peptides may date back to the time of the emergence of early chordates. Based on these new findings, this review highlights the evolution of GnIH peptide structure and its function.

## Discovery of GnIH in the brain as a novel key factor regulating reproduction

A neuropeptide possessing C-terminal Arg-Phe-NH_2_ motif (RF-amide peptide) was first identified in the ganglia of venus clam *Macrocallista nimbosa* (Price and Greenberg, 1977). Since important functions of RF-amide peptides as neurotransmitters, neuromodulators or peripheral hormones were revealed in invertebrates (Greenberg and Price, 1992), there have been attempts to identify RF-amide peptides in the central nervous system of vertebrates. Tsutsui et al. (2000) discovered a novel RF-amide peptide from brains of the Japanese quail *Coturnix japonica* (Tsutsui et al., 2000). An immunohistochemical study showed that the GnIH-immunoreactive cell bodies exist in the paraventricular nucleus (PVN) and their fibers project to the median eminence where neurochemicals that regulate the anterior pituitary are released (Tsutsui et al., 2000). Therefore, this RF-amide peptide was considered to regulate the function of anterior pituitary in quail (Tsutsui et al., 2000). Indeed this novel RF-amide peptide inhibited gonadotropin release from the cultured quail pituitary and thus the RF-amide peptide was termed GnIH (Tsutsui et al., 2000).

Quail GnIH is a dodecapeptide having a C-terminal RF-amide motif, SIKPSAYLPLRF-amide (Table 1). The sequence of the five amino acids at the C-terminal of quail GnIH was identical to chicken LPLRF-amide that was isolated as a first RF-amide peptide in vertebrates (Dockray et al., 1983). This chicken LPLRF-amide may be a fragment of chicken GnIH (for reviews, see Tsutsui, 2009; Tsutsui et al., 2010a,b). In 2001, a cDNA encoding GnIH precursor polypeptides was identified in quail (Satake et al., 2001). Now GnIH cDNAs have been identified in several avian species, such as chickens, sparrows, starlings and zebra finches (for reviews, see Tsutsui, 2009; Tsutsui et al., 2010a,b). The GnIH precursor encodes one GnIH and two GnIH-related peptides (GnIH-RP-1 and GnIH-RP-2) that possess a conserved Leu-Pro-Xaa-Arg-Phe-NH_2_ (LPXRF-amide; X = L or Q) motif at their C-termini in all birds studied (Table 1). Thus, GnIH and related peptides are called LPXRF-amide peptides from a structural point of view (for reviews, see Tsutsui, 2009; Tsutsui et al., 2010a,b). GnIH was further isolated as an endogenous ligand in European starling *Sturnus vulgaris* (Ubuka et al., 2008) and zebra finch *Taeniopygia guttata* (Tobari et al., 2010) and endogenous GnIH-RP-2 was also identified in quail (Table 1; Satake et al., 2001).

**Table 1:** **Amino acid sequences of GnlHs in chordates**.

	**Animal**	**Name**	**Sequence**	**References**
Mammals	Human	RFRP-1	**MPHSFANLPLRFa**	Ubuka et al., 2009b
		RFRP-3	**VPNLPQRFa**	Ubuka et al., 2009b
	Macaque	RFRP-1^*^	**MPHSVTNLPLRFa**	Ubuka et al., 2009a
		RFRP-3	**SGRNMEVSLVRQVLNLPQRFa**	Ubuka et al., 2009a
	Bovine	RFRP-1	**SLTFEEVKDWAPKIKMNKPVVNKM**	Fukusumi et al., 2001
			**PPSAANLPLRFa**	
		RFRP-3	**AMAHLPLRLGKNREDSLSRWVPNLPQRFa**	Yoshida et al., 2003
	Horse	RFRP-3^*^	**IPNLPQRFa**	Thorson et al., 2014
	Rat	RFRP-1^*^	**SWFQELKDWGAKKDIKMSPAPANKVPHS**	Ukena et al., 2002
			**AANLPLRFa**	
		RFRP-3	**ANMEAGTMSHFPSLPQRFa**	Ukena et al., 2002
	Siberian	RFRP-1	**SPAPANKVPHSAANIiPLRFa**	Ubuka et al., 2012a
	hamster	RFRP-3	**TLSRVPSLPQRFa**	Ubuka et al., 2012a
Birds	Quail	GnlH	**SIKPSAYLPLRFa**	Tsutsui et al., 2000
		GnIH-RP-1^*^	**SLNFEEMKDWGSKNFMKVNTPTVN**	Satake et al., 2001
			**KVPNSVANLPLRFa**	
		GnIH-RP-2	**SSIQSLLNLPQRFa**	Satake et al., 2001
	Chicken	GnlH^*^	**SIRPSAYLPLRFa**	Ikemoto and Park, 2005
		GnIH-RP-1^*^	**SLNFEEMKDWGSKNFLKVNTPTVNKV**	Ikemoto and Park, 2005
			**PNSVANLPLRFa**	
		GnIH-RP-2^*^	**SSIQSLLNLPQRFa**	Ikemoto and Park, 2005
	White-crowned sparrow	GnlH^*^	**SIKPFSNLPLRFa**	Osugi et al., 2004
		GnIH-RP-1^*^	**SLNFEEMEDWGSKDIIKMNPFTASKMPNS**	Osugi et al., 2004
		GnIH-RP-2^*^	**VANLPLRFa**	
			**SPLVKGSSQSLLNLPQRFa**	Osugi et al., 2004
	European starling	GnlH	**SIKPFANLPLRFa**	Ubuka et al., 2008
		GnIH-RP-1^*^	**SLNFDEMEDWGS KD IIKMNPFTVS**	Ubuka et al., 2008
			**KMPNS VANL PLRFa**	
		GnIH-RP-2^*^	**GSSQSLLNLPQRFa**	Ubuka et al., 2008
	Zebra finch	GnlH	**SIKPFSNLPLRFa**	Tobari et al., 2010
		GnIH-RP-1^*^	**SLNFEEMEDWRSKDIIKMNPFAASKMPN**	Tobari et al., 2010
			**SVANLPLRFa**	
		GnIH-RP-2^*^	**SPLVKGSSQSLLNLPQRFa**	Tobari et al., 2010
Reptiles	Anole lizard	GnlH^*^	**SIKPAANLPLRFa**	EN SACAG00000013069
		GnIH-RP-1^*^	**SMDLESMNDWELNKIIRRTTPEMKKMA**	EN SACAG00000013069
			**HAAVNLPLRFa**	
		GnIH-RP-2^*^	**APDVQSLSRSLANLPQRFa**	EN SACAG00000013069
	Chinese softshell turtle	GnlH^*^	**IIKPVANLPLRFa**	EN SPSIG00000017952
		GnIH-RP-1^*^	**SLNFEELKDWGSKNIIKMSTPTVNKM**	EN SPSIG00000017952
			**PNSVANLPLRFa**	
		GnIH-RP-2^*^	**TPFVKTSSQLFPNLPQRFa**	EN SPSIG00000017952
Amphibians	Bullfrog	fGRP/R-RFa	**SLKPAANLPLRFa**	Chartrel et al., 2002; Koda et al., 2002
		fGRP-RP-1	**SIPNLPQRFa**	Ukena et al., 2003
		fGRP-RP-2	**LSGKTKVQSMANLPQRFa**	Ukena et al., 2003
		fGRP-RP-3	**QYTNHFVHSLDTLPLRFa**	Ukena et al., 2003
	Red-bellied newt	nLPXRFa-1	**SVPNLPQRFa**	Chowdhury et al., 2011
		nLPXRFa-2	**MPHASANLPLRFa**	Chowdhury et al., 2011
		nLPXRFa-3	**SIQPLANLPQRFa**	Chowdhury et al., 2011
		nLPXRFa-4	**APSAGQFIQTLANLPQRFa**	Chowdhury et al., 2011
Teleost fish	Goldfish	gfLPXRFa-1^*^	**PTHLHANLPLRFa**	Sawada et al., 2002b
		gfLPXRFa-2^*^	**AKSNINLPQRFa**	Sawada et al., 2002b
		gfLPXRFa-3	**SGTGLSATLPQRFa**	Sawada et al., 2002b
	Medaka	mdLPXRFa-1^*^	**PLHMHANMPLRFa**	XM_004073848
		mdLPXRFa-2^*^	**VSNSSPNMPQRFa**	XM_004073848
		mdLPXRFa-3^*^	**EAPSPVLPQRFa**	XM_004073848
	Grass puffer	gpLPXRFa-1^*^	**SLDMERINIQVSPTSGKVSLPTIVRLYPT**	Shahjahan et al., 2011
			**LQPHHQHVN**	
			**-MPMRFa**	
		gpLPXRFa-2^*^	**DGVQGGDHVPNLNPNMPQRFa**	Shahjahan et al., 2011
		gpRYa^*^	**SWKVIRLCEDCSKVQGVLKHQVRYa**	Shahjahan et al., 2011
Agnathans	Sea lamprey	lLPXRFa-la	**SGVGQGRSSKTLFQPQRFa**	Osugi et al., 2012
		lLPXRFa-lb	**AALRSGVGQGRSSKTLFQPQRFa**	Osugi et al., 2012
		lLPXRFa-2	**SEPFWHRTRPQRFa**	Osugi et al., 2012
Protochordates	Amphioxus	PQRFa-1	**WDEAWRPQRFa**	Osugi et al., 2014
		PQRFa-2	**GDHTKDGWRPQRFa**	Osugi et al., 2014
		PQRFa-3	**GRDQGWRPQRFa**	Osugi et al., 2014

*Ensembl accession numbers or Genbank accession numbers are referred to for reptile GnlHs or medaka GnlHs*.

* *Indicates putative peptides*.

## Unity and diversity of GnIH structure in chordates

**Figure 1:**
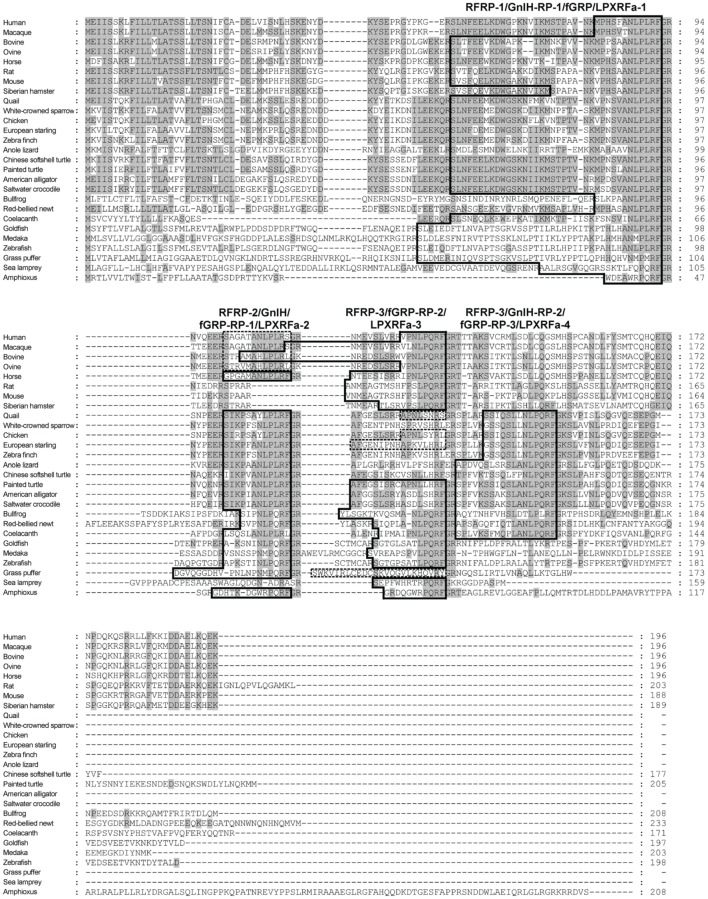
**Comparison of GnIH precursor amino acid sequences in representative species of chordates**. The identical amino acids are shaded. The peptide coding regions are boxed. The precursors of human, macaque, bovine, ovine, quail, chicken, starling, and grass puffer encode putative peptides with C-terminal RS-amide, RL-amide, or RY-amide motifs that are boxed by broken lines.

A mammalian GnIH, also known as RFamide-related peptide (RFRP), orthologous gene has been identified by using *in silico* analysis (Hinuma et al., 2000). The mammalian GnIH cDNAs encoded two GnIH peptides (RFRP-1 and -3) (Table 1 and Figure 1). Human, macaque, bovine and ovine precursor cDNAs also encoded a putative GnIH-like peptide that possesses a C-terminal LPLRSamide or LPLRLamide motif, which was named RFRP-2. However, rodent GnIH precursors lost RFRP-2 (Figure 1) (for reviews, see Tsutsui, 2009; Tsutsui et al., 2010a,b). Interestingly, the putative horse RFRP-2 possesses a C-terminal LPLRFamide motif (Figure 1) (Thorson et al., 2014). The mammalian GnIHs, RFRP-1 and/or RFRP-3, were identified as mature peptides in the brains of bovine *Bos taurus* (Fukusumi et al., 2001; Yoshida et al., 2003), rat *Rattus norvegicus* (Ukena et al., 2002), Siberian hamster *Phodopus sungorus* (Ubuka et al., 2012a), monkey *Macaca mulatta* (Ubuka et al., 2009a), and human *Homo sapiens* (Table 1; Ubuka et al., 2009b). GnIH and related peptides identified in birds and mammals have a conserved LPXRF-amide motif at the C-terminus (Table 1).

In reptiles, a putative GnIH gene was found in the Ensembl genome database of anole lizard, Chinese softshell turtle and painted turtle (Table 1; Figure 1). Recently, the crocodilian genome project was completed and the genome data of American alligator *Alligator mississippiensis* and saltwater crocodile *Crocodylus porosus* are available on the website of Crocodilian Genome Project (St. John et al., 2012; http://crocgenomes.org/). The putative GnIH gene was found in the genome data of crocodilians by using a tblastn program and exon-intron calculation based on the GT-AG rule (Figure 1; Mount, 1982). The putative reptilian GnIH peptides possess a C-terminal LPXRF-amide (X = L or Q) motif and showed a high sequence similarity with avian GnIH peptides that reflects a close phylogenetic position between birds and reptiles (Table 1; Figure 1).

In amphibians, a GnIH peptide was identified in the hypothalamus of bullfrog *Rana catesbeiana* and named frog growth hormone-releasing peptide (fGRP) (Table 1; Koda et al., 2002). cDNA cloning revealed that the precursor polypeptide encodes four GnIH peptides (fGRP, fGRP-RP-1, -RP-2, and RP-3) (Sawada et al., 2002a). fGRP-RP-1, -RP-2, and RP-3 were also identified as mature peptides (Table 1; Ukena et al., 2003). fGRP was independently isolated from the European green frog *Rana esculenta* and named *Rana* RFamide (R-RFa) (Chartrel et al., 2002). A GnIH cDNA was also cloned from the Japanese red-bellied newt, an urodele amphibian (Chowdhury et al., 2011). The deduced precursor encoded four GnIH peptides (nLPXRFa-1, -2, -3, -4), and these peptides were identified as mature peptides from the brain extracts (Table 1; Chowdhury et al., 2011). The rate of amino acid substitution or deletion may have been lower in the lineage of amphibians compared with other vertebrates, resulting in the conservation of four LPXRF-amide (X = L or Q) peptides encoded in the precursor (Figure 1).

**Figure 2:**
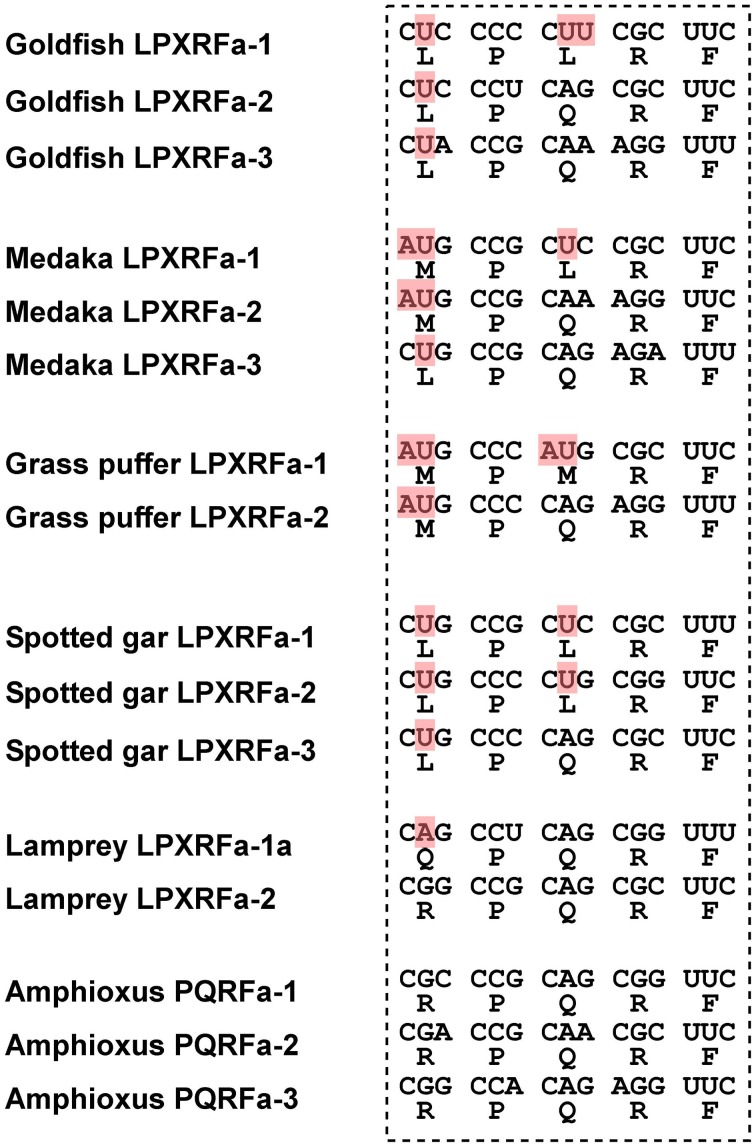
**Comparison of the C-terminal five amino acid sequences of GnIH peptides and their corresponding codons in teleost fish (goldfish, medaka, grass puffer, spotted gar), agnathan (lamprey), and protochordate (amphioxus)**. The nucleotides that have been modified during evolution are shaded in red.

In teleost fish, a GnIH cDNA encoding three peptides (gfLPXRFa-1, -2, and -3) which have C-terminal LPXRF-amide (X = L or Q) sequences was cloned from the brain of goldfish *Carassius auratus*, and one peptide (gfLPXRFa-3) was identified as a mature peptide (Table 1; Sawada et al., 2002b). A GnIH gene was also identified in the grass puffer *Takifugu niphobles*. The grass puffer GnIH precursor contained two putative GnIH peptides which have C-terminal MPMRF-amide or MPQRF-amide sequences and one possible RY-amide peptide (Table 1; Shahjahan et al., 2011). The medaka GnIH precursor contained two putative GnIH peptides which have C-terminal MPLRF-amide or MPQRF-amide sequences and one LPQRF-amide peptide (Table 1; XM_004073848). Therefore, Leu, Met, and Glu are substituted by each other in some fish species. The CUG codon encoding Leu can be mutated to AUG encoding Met by a single nucleotide substitution. Similarly, a single nucleotide substitution in the codon encoding Glu (CAA and CAG) can produce CUA and CUG encoding Leu. Thus, nucleotide substitutions in the codon encoding the third and the fifth amino acids from the C-terminal may have occurred in some fish species, such as medaka and grass puffer (Figure 2). We further searched for GnIH-like sequences in the genome database of phylogenetically important fish, such as the elephant shark, skate, and spotted gar. A partial GnIH-like sequence was found in the Ensembl genome database of the spotted gar (chromosome LG11, nt 40715843 to nt 40716142, reverse strand). The C-terminal motifs of spotted gar GnIH-like peptides were LPLRF or LPQRF and their codons were similar to those of other fish (Figure 2). On the other hand, we could not find any GnIH-like sequence in the elephant shark genome database (http://esharkgenome.imcb.a-star.edu.sg/) and the skate genome database (http://skatebase.org/). Further researches are needed to clarify the presence of GnIH in cartilaginous fish.

Recently, we have identified a GnIH orthologous gene by using synteny analysis and cDNA cloning in lamprey, one of the most basal vertebrates (Figure 1; Osugi et al., 2012). Mature lamprey GnIH peptides were also identified by using immunoaffinity purification and mass spectrometry (Table 1; Osugi et al., 2012). The lamprey GnIH peptides possessed a C-terminal QPQRF-amide or RPQRF-amide motif and the third or the fifth Leu from the C-terminal was not conserved (Table 1; Osugi et al., 2012). The codon encoding Glu in QPQRF-amide and RPQRF-amide were all CAG, and the codon encoding the first Arg in RPQRF-amide was CGG (Figure 2). The codon CAG or CGG can be mutated to CUG encoding Leu by a single nucleotide substitution. Therefore, the fifth and the third Leu from the C-terminal may have appeared and conserved after the emergence of gnathostomes.

We further searched for GnIH in amphioxus, the most basal chordate, to investigate the evolutionary origin of GnIH. A novel gene encoding RF-amide peptides and mature peptides were identified in the amphioxus *Branchiostoma japonicum* by using genome database search in *Branchiostoma floridae*, cDNA cloning and immunoaffinity purification (Table 1; Figure 1; Osugi et al., 2014). The identified amphioxus RF-amide peptides possessed a C-terminal RPQRF-amide motif that was identical to the C-terminal of lamprey LPXRFa-2 (Table 1). The codon encoding the first Arg in RPQRF-amide was CGC, CGA, or CGG, which can be mutated to CUC, CUA, or CUG encoding Leu by a single nucleotide substitution, respectively (Figure 2). The codon encoding the third Glu in RPQRF-amide was CAG or CAA, which can be mutated to CUG or CUA encoding Leu by a single nucleotide substitution, respectively (Figure 2). Accordingly, nucleotide mutations may have occurred at the codon encoding the first Arg and the third Glu in RPQRF-amide during the course of vertebrate evolution, resulting in the C-terminal LPQRF-amide or LPLRF-amide motif of GnIH peptides in gnathostomes.

## Unity and diversity of GnIH function in vertebrate reproduction

The function of GnIH and related peptides are summarized in Table 2. As described above, the gonadotropin inhibiting effect of GnIH was first demonstrated in the quail pituitary *in vitro* (Tsutsui et al., 2000). An *in vivo* study further revealed that GnIH inhibits the release and expression of gonadotropins in quail (Ubuka et al., 2006). In addition to the direct effect of GnIH on the pituitary, GnIH also inhibited GnRH-induced elevation in plasma LH in song sparrow (Osugi et al., 2004). The close proximity of GnIH immunoreactive fibers to GnRH neurons and the expression of GnIH receptor in GnRH neurons support the effect of GnIH on GnRH neurons in birds (Ubuka et al., 2008). To investigate the mode of action of GnIH in birds, the receptor for GnIH was identified in quail brain (Yin et al., 2005). GnIH receptor (GnIH-R) is a G-protein-coupled receptor, also known as GPR147, and it was expressed in the pituitary and several brain regions including diencephalon (Yin et al., 2005). GnIH-R showed high affinities to GnIH, GnIH-RPs, and RFRPs, which have LPXRF-amide (X = L or Q) motif at their C-termini (Yin et al., 2005). Non-amidated GnIH failed to bind the receptor, suggesting that the C-terminal LPXRF-amide (X = L or Q) motif is responsible for its binding to GnIH-R (Yin et al., 2005). It was further demonstrated that GnIH-R couples to G_α*i*_ and GnIH inhibits GnRH-induced cAMP responsive element (CRE) activation in the chicken, suggesting that GnIH regulates GnRH signaling by inhibiting cAMP signaling pathway (Bédécarrats et al., 2009; Shimizu and Bédécarrats, 2010). From the viewpoint of the behavioral regulation, intracerebroventricularly (ICV) administered GnIH inhibited reproductive behavior of female white-crowned sparrows (Bentley et al., 2006). By using the RNAi technique, it was shown that GnIH regulates aggressive and sexual behaviors in male white-crowned sparrow or quail (Ubuka et al., 2012b, 2013b). Recently, it was further demonstrated that GnIH inhibits socio-sexual behavior of male quail by increasing neuroestrogen synthesis in the hypothalamus (Ubuka et al., 2014). At the peripheral level, GnIH decreased plasma testosterone concentration, induced testicular apoptosis and decreased spermatogenic activity in adult male quail, suggesting a direct action of GnIH at the testis or an action *via* reduced gonadotropin secretion (Ubuka et al., 2006). In addition, GnIH also reduced the testicular weight in immature birds, suggesting that GnIH is involved in gonadal development and maintenance (Ubuka et al., 2006). Taken together, GnIH acts as an inhibitory neuropeptide and exerts multiple effects on the reproductive systems in the brain as well as peripheral organs.

**Table 2:** **Functions of GnlH and related peptides in vertebrates**.

**Animal**	**Name**	**Function**	**References**
Syrian hamster	GnlH	Inhibition of LH release	Kriegsfeld et al., 2006
Siberian hamster	RFRP-1 and-3	Inhibition or stimulation of LH release	Ubuka et al., 2012a
Rat	RFRP-3	Inhibition of LH secretion	Johnson et al., 2007
		Inhibition of GnRH-elicited LH release	Murakami et al., 2008
Mouse	RFRP-3	Inhibition of the firing rate of GnRH neurons	Ducret et al., 2009
Ovine	RFRP-3	Inhibition of GnRH-elicited gonadotropin release	Clarke et al., 2008
		Inhibition of gonadotropin secretion	Sari et al., 2009
Bovine	RFRP-3	Inhibition of LH release	Kadokawa et al., 2009
Pig	RFRP-3	Inhibition of GnRH secretion	Li et al., 2013
Quail	GnlH	Inhibition of LH release	Tsutsui et al., 2000
		Inhibition of gonadotropin secretion	Ubuka et al., 2006
		Inhibition of socio-sexual behavior	Ubuka et al., 2014
		Inhibition of plasma testosterone concentration	Ubuka et al., 2014
		Reduction of testicular weight	Ubuka et al., 2006
Sparrow	GnlH	Inhibition of GnRH-induced elevation in plasma LH	Osugi et al., 2004
		Inhibition of reproductive behavior	Bentley et al., 2006
		Inhibition of aggressive and sexual behaviors	Ubuka et al., 2012b, 2013b
		Inhibition of socio-sexual behavior	Ubuka et al., 2014
Chicken	GnlH	Inhibition of GnRH-induced CRE activation	Bédécarrats et al., 2009; Shimizu and Bédécarrats, 2010
Sockeye salmon	gfLPXRFa-1	Stimulation of gonadotropin and GH release	Amano et al., 2006
	gfLPXRFa-2	Stimulation of gonadotropin and GH release	Amano et al., 2006
	gfLPXRFa-3	Stimulation of gonadotropin and GH release	Amano et al., 2006
Grass puffer	gfLPXRFa-1	Stimulation of gonadotropin expression	Shahjahan et al., 2011
Goldfish	zfLPXRFa	Inhibition of plasma LH concentration	Zhang et al., 2010
Goldfish	gfLPXRFa-2	Inhibition of sGnRH and FSH expression	Qi et al., 2013
Goldfish	gfLPXRFa-2	Inhibition of LH expression	Qi et al., 2013
Goldfish	gfLPXRFa-3	Inhibition of sGnRH and FSH expression	Qi et al., 2013
Goldfish	gfLPXRFa-3	Inhibition of GnRH-stimulated gonadotropin synthesis	Qi et al., 2013
Goldfish	GnlH	Inhibition or stimulation of gonadotropin secretion	Moussavi et al., 2012, 2013
Lamprey	LPXRFa-2	Stimulation of gonadotropin expression	Osugi et al., 2012

In mammals, two GnIH peptides (RFRP-1 and RFRP-3) are encoded in the precursor. As in birds, RFRP-3 inhibited gonadotropin synthesis and/or release in various mammalian species (Kriegsfeld et al., 2006; Johnson et al., 2007; Clarke et al., 2008; Murakami et al., 2008; Kadokawa et al., 2009; Sari et al., 2009; Ubuka et al., 2012a). In addition, immunoreactive GnIH fibers were in close proximity to GnRH neurons in the hypothalamus of human, monkey, sheep, rat and hamster (Kriegsfeld et al., 2006; Johnson et al., 2007; Smith et al., 2008; Ubuka et al., 2009a,b, 2012a). Expression of GnIH-R was also observed in GnRH neurons (Rizwan et al., 2012; Ubuka et al., 2012a). Consistent with histochemical studies, RFRP-3 was shown to inhibit the firing rate of GnRH neurons in mice (Ducret et al., 2009). In pig, RFRP-3 also inhibited the synthesis and release of GnRH (Li et al., 2013). Recently, it was found that ICV administration of RFRP-1 also inhibits gonadotropin release in hamsters (Ubuka et al., 2012a). Therefore, both RFRP-1 and RFRP-3 may act as GnIH in mammals. Regarding the signal transduction mechanisms in mammals, it was demonstrated that RFRP-3 reduces GnRH-stimulated cytoplasmic calcium response and extracellular signal-regulated kinase (ERK) phosphorylation in sheep pituitary (Clarke et al., 2008; Sari et al., 2009). The detailed mechanisms were further investigated using mouse gonadotrope cell line (LβT2 cells). It was revealed that the inhibitory action of mouse GnIHs (RFRPs) on gonadotropin gene expression is mediated by an inhibition of adenylate cyclase (AC)/cAMP/cAMP-dependent protein kinase A (PKA)-dependent ERK pathway (Son et al., 2012). These studies in mammals suggest that the inhibitory mechanism of GnIH on the reproductive system is conserved among mammalian and avian animals. In Siberian hamsters, the expression of GnIH decreased in short day conditions by the action of pineal melatonin (Ubuka et al., 2012a). Siberian hamster GnIHs (RFRP-1 and RFRP-3) stimulated LH release in short day conditions and inhibited LH release in long day conditions (Ubuka et al., 2012a). Because Siberian hamsters are long day breeders and short day conditions represent an inhibitory photoperiod, GnIH may sustain appropriate concentration of LH in short day condition. Taken together, GnIH may have acquired a stimulatory function in the lineage of photoperiodic mammals to optimize their reproductive activities according to the season.

In teleost fish, functional diversity was observed compared to mammals and birds. Goldfish GnIHs (gfLPXRFa peptides) stimulated release of gonadotropins and growth hormone (GH) in Sockeye salmon *Oncorhynchus nerka* (Amano et al., 2006). In grass puffer, the expression of GnIH and GnIH-R mRNA were increased during the spawning season in the brain and pituitary (Shahjahan et al., 2011). In addition, goldfish GnIH (gfLPXRFa-1) stimulated the expression of gonadotropin mRNAs in the pituitary in grass puffer, suggesting that GnIH may be involved in the stimulation of reproductive axis in grass puffer (Shahjahan et al., 2011). In contrast to the stimulatory effects of fish GnIH, zebrafish GnIH decreased serum LH level in goldfish *in vivo* (Zhang et al., 2010). Similarly, goldfish GnIHs (gfLPXRFa-2 and -3) decreased salmon GnRH and FSHβ mRNA levels and gfLPXRFa-2 decreased LHβ mRNA levels in goldfish *in vivo* (Qi et al., 2013). Although single administrations of goldfish GnIHs (gfLPXRFa-2 and -3) showed no effect on gonadotropin synthesis in the primary culture of goldfish pituitary cells, gfLPXRFa-3 inhibited GnRH-stimulated LHβ and FSHβ synthesis (Qi et al., 2013). According to the maturational status of goldfish, goldfish GnIH exerted both stimulatory and inhibitory effects on the expression of gonadotropin mRNAs and the serum LH level (Moussavi et al., 2012, 2013). The studies in teleost fish suggest that the functions of GnIH are diverse even within the teleost fish lineage. The action of GnIH also changes depending on the physiological conditions of the fish.

In agnathans, lamprey GnIH-immunoreactive fibers were in close proximity to GnRH-III neurons (Osugi et al., 2012). One of the GnIH peptides (lamprey LPXRFa-2) administered *in vivo* increased GnRH-III concentration in the brain and mRNA expression of gonadotropin β subunit in the pituitary (Osugi et al., 2012). These effects of GnIH are similar to that of some of teleost fish, suggesting that the stimulatory action of GnIH may have been conserved in several lineages of basal vertebrates.

## Evolutionary origin of GnIH and its ancestral structure and function

**Figure 3:**
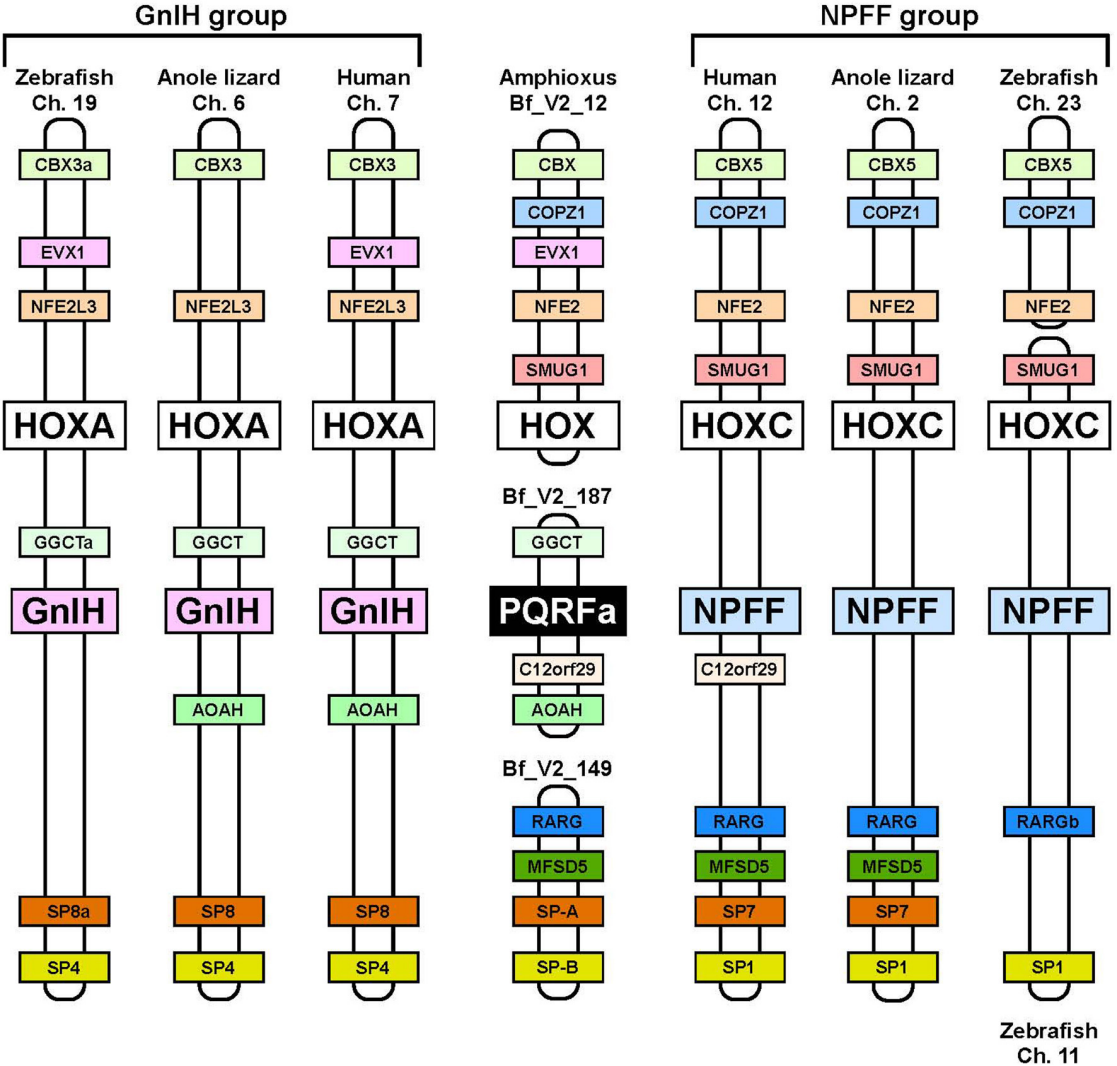
**Synteny analysis of the loci of amphioxus PQRF-amide peptide gene, GnIH gene, and NPFF gene**. The names of animals and chromosome or scaffold numbers are shown on the top or bottom of each chromosome or scaffold region. Orthologous genes are shown in a same color. The amphioxus PQRF-amide peptide gene, vertebrate GnIH genes and vertebrate NPFF genes are shown in black, pink, or blue boxes. HOX clusters are shown in white boxes. The conserved synteny region exists around the loci of amphioxus PQRF-amide peptide gene, GnIH gene and NPFF gene. The distance of the genome fragments analyzed were as follows: 15.08 Mbp (human Ch. 7), 17.24 Mbp (anole lizard Ch. 6), 18.46 Mbp (zebrafish Ch. 19), 1.09 Mbp (human Ch. 12), 3.91 Mbp (anole lizard Ch. 2), 40 Kbp (zebrafish Ch. 23), and 259.76 Mbp (zebrafish Ch. 11). Note that anole lizard HOXA and SP8 are on the scaffolds GL343275.1 and GL343212.1. The homology between these genome regions was identified based on the gene annotation in the Ensembl genome database. The homologous genes of amphioxus were searched by the blast program in the Joint Genome Institute.

**Figure 4:**
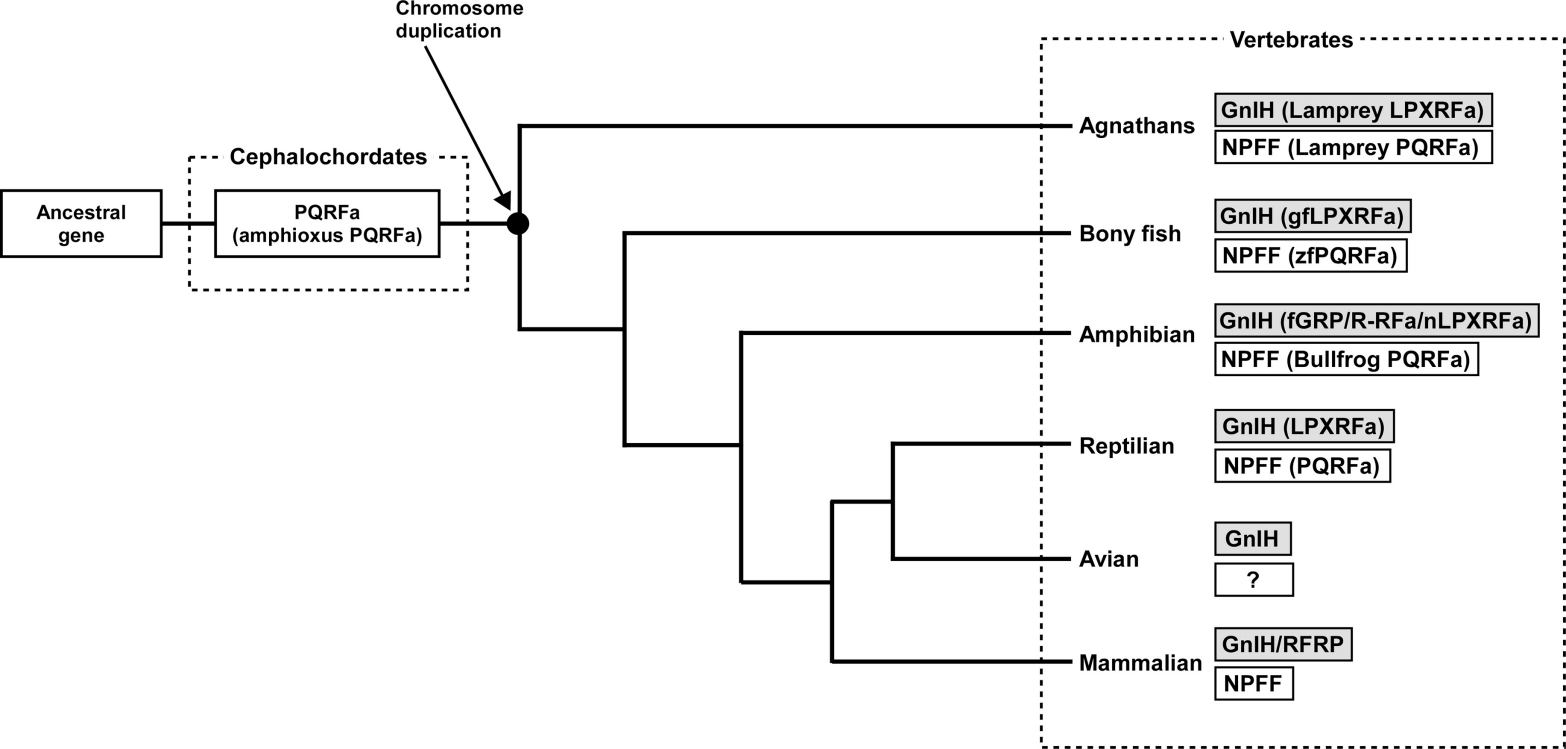
**Proposed evolutionary history of GnIH and NPFF genes**. GnIH and NPFF genes may have originated from a common ancestral gene. The amphioxus which has not experienced chromosome duplication has sustained an ancestral form of GnIH and NPFF genes. GnIH and NPFF genes may have evolved through chromosome duplication that has occurred at the beginning of vertebrate evolution. ?, unknown.

Neuropeptide FF (NPFF), also known as PQRF-amide peptide is a pain-modulatory neuropeptide and is considered to be a paralogous gene of GnIH. The C-terminal motifs of GnIH and NPFF that are important for the interaction with their receptors showed high sequence similarity (Osugi et al., 2006, 2011, 2012). The receptors for GnIH (NPFFR1; GPR147) and NPFF (NPFFR2; GPR74) also showed a high sequence similarity (Yin et al., 2005). In addition, the GnIH gene locates near the *HOXA* clusters and NPFF gene locates near the *HOXC* clusters on the chromosome (Figure 3; Ikemoto and Park, 2005; Osugi et al., 2012). The sequence similarity of GnIH and NPFF and a common belief that the *HOX* clusters have duplicated from a common ancestral gene during whole genome duplication events through vertebrate evolution (Venkatesh et al., 2007) led to the strong hypothesis that GnIH gene and NPFF gene have diverged from a common ancestral gene through chromosome duplication (Ikemoto and Park, 2005; Osugi et al., 2012, 2014). The presence of both GnIH and NPFF in agnathans, the most ancient vertebrates, further suggests that GnIH and NPFF genes have diverged before the emergence of vertebrates (Osugi et al., 2006, 2011, 2012). As described above, we identified a novel gene encoding RF-amide peptides and mature peptides in the amphioxus *Branchiostoma japonicum* (Table 1; Figure 1; Osugi et al., 2014). We further identified putative receptors for the identified amphioxus RF-amide peptides. Molecular phylogenetic analysis and synteny analysis indicated that these genes are closely related to GnIH and NPFF genes and their receptors of vertebrates (Figure 3; Osugi et al., 2014). The identified amphioxus RF-amide peptides inhibited forskolin induced cAMP signaling in the COS-7 cells transfected with one of the identified amphioxus RF-amide peptide receptors (Osugi et al., 2014). The study of amphioxus thus indicates that the identified protochordate RF-amide peptide gene is likely to be an ancestral form of both GnIH and NPFF. We could not find any GnIH-like peptide sequence in other early deuterostome genomes, such as *Ciona intestinalis*, sea urchin and acorn worm. Therefore, the origin of GnIH may date back to the time of the emergence of early chordates (Figure 4). The role of the ancestral GnIH may have been an inhibitory peptide and its functions may have been diversified in some lineage during the course of vertebrate evolution.

Currently, the following RF-amide peptide groups, namely, GnIH, NPFF, 26RF-amide peptide (26RFa)/pyroglutamylated RF-amide peptide (QRFP), prolactin-releasing peptide (PrRP), Kiss1, and Kiss2 are found in the brain of vertebrates (Tsutsui, 2009; Tsutsui et al., 2010b). Within these groups, GnIH and NPFF, and Kiss1 and Kiss2 are thought to be paralogous (Felip et al., 2009; Lee et al., 2009; Osugi et al., 2012, 2013, 2014; Pasquier et al., 2012). It is of interest to investigate the evolutionary relationship between RF-amide peptide groups, and search the common origin of these RF-amide peptides. Recent advances in genome database with powerful instruments, such as bioinformatic tools and next-generation sequencing, may enable us to analyze the genome data of various animals and answer to this question.

### Conflict of interest statement

The authors declare that the research was conducted in the absence of any commercial or financial relationships that could be construed as a potential conflict of interest.
